# Income level and antibiotic misuse: a systematic review and dose–response meta-analysis

**DOI:** 10.1007/s10198-021-01416-8

**Published:** 2021-11-30

**Authors:** Narmeen Mallah, Nicola Orsini, Adolfo Figueiras, Bahi Takkouche

**Affiliations:** 1 Department of Global Public Health, Karolinska Institutet, Stockholm, Sweden; 2 Department of Preventive Medicine, University of Santiago de Compostela, Santiago de Compostela, Spain; 3 Centro de Investigación Biomédica en Red de Epidemiología y Salud Pública (CIBER-ESP), Madrid, Spain; 4 Health Research Institute of Santiago de Compostela (IDIS), Santiago de Compostela, Spain; 5 Department of Preventive Medicine, Faculty of Medicine, University of Santiago de Compostela, 15782 Santiago de Compostela, Spain

**Keywords:** Income, Antibiotics, Misuse, Meta-analysis, Dose–response

## Abstract

**Objectives:**

To quantify the association between income and antibiotic misuse including unprescribed use, storage of antibiotics and non-adherence.

**Methods:**

We identified pertinent studies through database search, and manual examination of reference lists of selected articles and review reports. We performed a dose–response meta-analysis of income, both continuous and categorical, in relation to antibiotic misuse. Summary odds ratios (ORs) and their 95% confidence intervals (CIs) were estimated under a random-effects random effects model.

**Results:**

Fifty-seven studies from 22 countries of different economic class were included. Overall, the data are in agreement with a flat linear association between income standardized to socio-economic indicators and antibiotic misuse (OR per 1 unit increment = 1.00, *p*-value = 0.954, *p*-value non-linearity = 0.429). Data were compatible with no association between medium and high income with general antibiotic misuse (OR 1.04; 95% CI 0.89, 1.20 and OR 1.03; 95% CI 0.82, 1.29). Medium income was associated with 19% higher odds of antibiotic storage (OR 1.19; 95% CI 1.07, 1.32) and 18% higher odds of any aspect of antibiotic misuse in African studies (OR 1.18; 95% CI 1.00, 1.39). High income was associated with 51% lower odds of non-adherence to antibiotic treatment (OR 0.49; 95% CI 0.34, 0.60). High income was also associated with 11% higher odds of any antibiotic misuse in upper-middle wealth countries (OR 1.11; 95% CI 1.00, 1.22).

**Conclusions:**

The association between income and antibiotic misuse varies by type of misuse and country wellness. Understanding the socioeconomic properties of antibiotic misuse should prove useful in developing related intervention programs and health policies.

## Introduction

The misuse of antibiotics is defined as the intake of these drugs without medical advice (self-prescription) or their use when prescribed by the physician but without compliance with the physician’s instructions for treatment regimen in terms of timing, dosage and duration [1, 2]. It is a salient problem worldwide, irrespective of the country economy and wealth. Antibiotic misuse has led to antibiotic resistance, a universal public health problem with high socioeconomic and clinical burdens. Different systematic reviews and meta-analyses reported the high prevalence of antibiotic misuse. In their study, Morgan et al. reviewed publications from five continents and concluded that the use of antibiotics without prescription is wide-reaching and accounts for 19 to 100% of antibiotic use outside Northern Europe and North America [3]. Gualano et al. also reported that almost half of the individuals stop taking antibiotics upon improvement [4]. Another review estimated that the mean use of leftover antibiotics worldwide is 29%, and that of compliance with antibiotic therapy is only 62% [5]. A recent meta-analysis of studies from low- and middle- income countries found that the pooled prevalence of non-prescribed use of antibiotics is considerably high (78%) in these countries [6]. Antibiotic misuse is also frequent in high- income countries, including the United States where the prevalence of antibiotic use without prescription is as high as 66% in some instances, and that of storage of antibiotics for future use ranges between 14 and 48% [7].

Antibiotic resistance causes at least 700,000 annual deaths worldwide [8], more than 35,000 in the United States alone [9]. A similar record is registered in Europe [10]. The impact of antibiotics resistance on the economy is also expanding with disturbing figures [11]. By 2050, the annual mortality rate from antibiotic resistance is projected to exceed that of major causes of death like cancer and diabetes [8], and the provoked economic shortfalls will be as large as that of the 2008–2009 global financial crisis [12].

Several determinants of antibiotic misuse have been identified. These are mainly sociodemographic, including female gender, young adults and elderly, low educational level, difficult access to the healthcare system, unaffordability of the cost of physicians visit and accessibility to antibiotics [7, 13, 14].

In 2012, a narrative review report about self-medication with antibiotics in developing countries analysed data of five studies and concluded that middle income is associated with antibiotic misuse [15]. Studies that evaluated the association of income with antibiotic misuse showed divergent results. Some studies reported up to six-fold increased odds of misuse in high- income individuals [16, 17], while other studies did not find any association [18–20], or detected lower odds of misuse [21, 22]. It is also unclear whether the association between income and antibiotic misuse holds at different social classes and in regions with different levels of access to healthcare and in which regulations about antibiotic dispensing might vary. To the best of our knowledge, there is no meta-analysis that evaluates the association of income with antibiotic misuse worldwide.

To address this gap, we aimed in this study to carry out a meta-analysis of the association of income with antibiotic misuse. We present analyses standardized for socio-economic indicators.

## Materials and methods

PRISMA guidelines were followed for the conduct and reporting of this meta-analysis, and the study protocol was registered in the PROSPERO database (ID: *number deleted for blinding purposes*). The outcome, antibiotic misuse, was defined as the use or purchase of non-prescribed antibiotics to treat oneself or another person, storage of leftover antibiotics, or nonadherence to the physicians’ instructions regarding the dosage, timing and treatment duration. Storage of antibiotics facilitates access to them and therefore is the first step towards their use without prescription [23].

### Literature search and study selection

Medline, EMBASE, Conference Proceedings Citation Index-Science, the Open Access Theses and Dissertations, and the five regional bibliographic databases of the World Health Organization (WHO) were searched since their inception until January 2021. The following search syntax was applied in Medline: (Socioeconomic Factors OR income) AND (antibiotic*) AND ((drug storage [MeSH]) OR (compliance) OR (adherence) OR (Nonprescription Drugs/administration & dosage* [MeSH]) OR (misuse) OR (irrational use) OR (left-over)) and adapted for the other databases. We complemented our search by using free text words as follows: antibiotics AND (misuse OR "unprescribed use" OR leftover OR "adherence to treatment") AND (income OR "socioeconomic status" OR "socioeconomic level"). The reference lists of related reviews [3–7, 13–15, 24, 25] and those of included studies were manually checked to supplement database searches. The search was carried out without any language or date restrictions.

Studies that met the following criteria were included: (1) reporting at least two levels of income with defined boundaries as an exposure, and (2) providing Odds Ratio (OR) or Risk Ratio (RR) and their 95% Confidence Interval (CI) as a measurement of the association of income and misuse of antibiotics by the general population, or sufficient data for their calculation.

### Data extraction and synthesis

From each included study, we extracted: (1) *general study characteristics*: author’s last name and year of publication, study period, participants characteristics (age and gender), and country where the study took place, (2) *exposure*: levels of monthly income, (3) *measures of association*: for each income level: number of subjects who practiced antibiotic misuse, total sample size, adjusted ORs and 95%CIs, and restriction, adjustment, or matching variables. When adjusted ORs were not provided, the crude estimates were registered, and (4) *Type of antibiotic misuse*: use without prescription, non-adherence, and storage of antibiotic leftover. When data was were provided for more than one type of antibiotic misuse, we extracted the data of all types of misuse. When the number of events of antibiotic misuse per income level was not available, we contacted the authors to request this information, but no reply was received [26–28]. We then deemed the number of events missing for those studies. We also inquired about the reference group used in a sub-analysis of one study [29], but due to lack of answer, we did not consider that subgroup.

We standardized the income to country-specific socio-economic indicators using two approaches. In the first approach, income was standardized to gross domestic product (GDP) *per capita* based on purchasing power parity (PPP) [30]. PPP is a currency conversion rate that is used to equalise the purchasing power of different monetary units. It allows to compare standards of living and economic productivity between countries [31]. In the second approach, the income level was standardized to the adjusted net national income *per capita*, expressed in US dollars [30]. The historical country-specific values of PPP, GDP *per capita* based on PPP, and adjusted net national income *per capita* were extracted from their specific portals in the World Bank [31–33].

Besides data reported in the studies, the classification of countries by economy [34], geographic distribution [35], and literacy rate [36] was obtained.

### Statistical analysis

Studies included in this meta-analysis presented income categorized into 2 to 6 levels, with an average of 3 levels. As an estimate of the dose, we used the midpoint assigned to an estimated contrast given the upper and lower boundaries of the income.

We carried out dose–response meta-analysis of income standardized to: (1) gross domestic product (GDP) *per capita* based on (PPP) and (2) adjusted net national income *per capita.*

The dose–response meta-analysis was performed using a one-stage mixed-effects model taking into account heterogeneity across studies [37, 38].

We first used a linear function to estimate a summary OR of antibiotic misuse associated with an increase of 1 unit in income. We next flexibly modelled income using restricted cubic splines with 3 knots fixed at 10th, 50th and 90th percentiles of its distribution. Tests of hypothesis about the regression coefficients of the dose–response model were conducted using a large sample Wald-type test. To facilitate tabular presentation of the summary odds ratios, we further categorized income into tertiles using the lowest as referent.

We stratified the dose–response analysis by type of antibiotic misuse (unprescribed use, storage of leftover, non-adherence); WHO geographic classification, country economy (low wealth, lower–middle wealth, upper–middle wealth and high-wealth); literacy rate (≥ 90%, < 90%); exposure ascertainment (use of pretested or validated questionnaire; untested questionnaire or not reported); comparability (control for age, sex, educational level and household size; incomplete control); and publication year (≤ 2015, > 2015). In 2015, WHO published the global action plan to combat the problem of antibiotic resistance [39].

### Quality appraisal

As all studies retrieved were eventually of cross-sectional nature, we appraised the quality of the studies using the Newcastle–Ottawa Scale for cross-sectional studies [40]. One point was given for the fulfilment of each of the following criteria: (1) well- defined target population; (2) reported response rate; (3) well described and appropriate statistical analysis; (4) justified sample size; (5) studies adjusted, matched or restricted for age, sex, educational level and household size; (6) use of previously tested or validated questionnaire; and (7) outcome ascertainment carried out using external assessment in addition to self-reporting. When information on a specific criterion was not given, it was graded with 0 point. The grades across items were then summed to obtain a quality score of a maximum of seven points. Two epidemiologists *(NM and AF)* carried out the quality assessment, and disagreements were resolved by referring to a third epidemiologist (BT).

### Publication bias

Publication bias was checked visually using funnel plot and formally through Egger’s test [41], and the trim-and-filltrim and fill method [42].

## Results

### Literature search and study

**Fig. 1:**
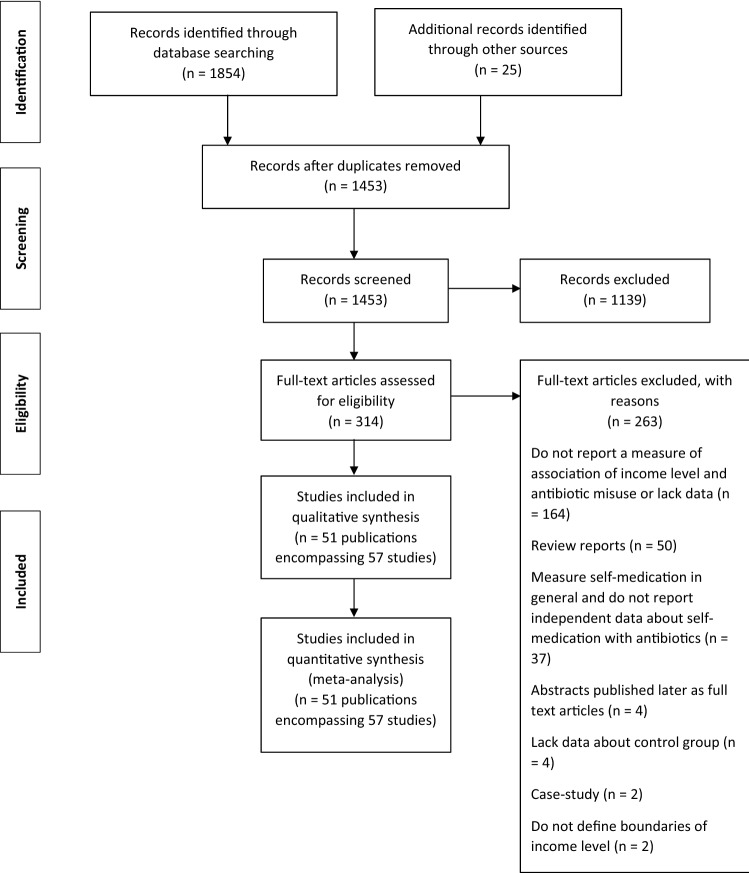
Flow diagram of the selection of studies about income level and misuse of antibiotics

Figure 1 represents the flow diagram of the selection of studies about income level and misuse of antibiotics. One thousand four hundred fifty-three publications were identified from the literature search, out of which 314 were selected for full- text review (Fig. 1). Fifty-one studies published between 2001 and 2021 met our inclusion criteria (Table 1). Five studies provided data for several types of misuse [20, 27, 28, 43, 44]. We treated each type of misuse as a separate study, making a total of 57 studies introduced in the dose–response analysis. All studies were of cross-sectional design. They involved a total population of 51,008 individuals from 22 countries and 18,094 events of antibiotic misuse. Forty-nine studies were published in English, one in Spanish [45] and one in Croatian [46].

**Table 1:** General characteristics of studies included the dose-–response meta-analysis of income level and antibiotic misuse

Author, Year	Country	Setting	Age (Years)	Sex	Outcome	Mean Income (USD)	Total N/level	Outcome/level	OR point estimate	Adjustment, restriction or matching variables
Moktan 2021[63]	India	Attendants of public hospital	18–90	M: 309 F: 195	Use without prescription	37.50	137	41	Reference category	Age, gender, educational level, marital status, public and private clinics, frequency of doctors’ consultation, family/friend influence (other family members self-medicating with antibiotics), symptoms (minor illness)
						112.51	185	59	1.10 (0.68–1.77)	
						225.01	129	52	1.58 (0.95–2.63)	
						375.01	53	19	1.31 (0.67–2.56)	
Bulabula 2020 [26]	South Africa	Pregnant women attending public hospital	Mean (SD): 29 (6.1)	F: 301	Use without prescription	49.50	–	–	Reference category	Age, gender, educational level, residential location, knowledge about antibiotics, attitudes towards antibiotics
						174.50	–	–	5.40 (0.90–29.90)	
						375.00	–	–	4.10 (0.80–19.40)	
						625.00	–	–	6.40 (1.20–35.20)	
Chen 2020 [43]	Mali	Medical university students	Mean (SD) 21.3 (2.4)	M:310 F:136	Storage of antibiotics	82.95	290	168	Reference category	Age
						506.50	114	77	1.51 (0.96–2.38)	
						1181.60	42	27	1.31 (0.67–2.56)	
					Use without prescription	82.95	290	73	Reference category	
						506.50	114	29	1.01 (0.62–1.67)	
						1181.60	42	19	2.46 (1.27–4.77)	
Elmahi 2020 [64]	Sudan	General population	≥ 18	M: 130 F: 116	Use without prescription	49.50	182	110	1.05 (0.59–1.87)	Age, pregnancy, current antibiotic use
						149.50	64	38	Reference category	
Mallah 2020 [59]	Lebanon	Children´s caregivers	≥ 18	M:276 F:1092	Any misuse practice	249.50	21	2	Reference category	Age, sex, educational level, area of residence, alcohol consumption, access to medical care facilities, and frequency of telephone medical consultation
						999.50	260	34	1.43 (0.32–6.41)	
						2000.00	223	17	0.78 (0.17–3.65)	
						3000.50	808	36	0.44 (0.10–1.98)	
Nusair 2020 [65]	Jordan	General population	0 to > 65	M: 674 F: 1169	Use without prescription	88.75	175	61	Reference category	Past month antibiotic use
						266.61	659	253	1.16 (0.82–1.65)	
						444.11	1042	458	1.47 (1.05–2.05)	
Rathish 2020 [18]	Sri Lanka	General population	Mean (SD): 36 (21)	M: 181 F: 203	Use without prescription	150.00	267	263	Reference category	NA
						450.00	117	111	2.15 (0.37–12.54)	
Xu 2020 [28]	China	Children´s caregivers	Parents with children < 13 years old	M: 1344 F: 4935	Use without prescription	377.50	–	–	Reference category	Age, gender, educational level, medical background, residential location
						1132.58	–	–	0.76 (0.57–1.03)	
						1887.58	–	–	0.81 (0.54–1.21)	
					Storage of antibiotics	377.50	–	–	Reference category	
						1132.58	–	–	1.03 (0.91–1.17)	
						1887.58	–	–	1.16 (0.99–1.36)	
Ateshim 2019 [66]	Eritrea	General population	Median (IQR): 37 (24)	M: 238 F: 339	Use without prescription	0.00	291	–	Reference category	Age, gender, educational level, marital status, occupational status, knowledge about antibiotics, attitudes towards antibiotics
						32.53	92	–	0.92 (0.54–1.56)	
						113.78	136	–	1.22 (0.78–1.19)	
						211.28	58	–	1.43 (0.75–2.73)	
Benameur 2019 [67]	Saudi Arabia	University students	Mean (SD): 20.96 (0.148)	M:166 F:69	Use without prescription	133.37	164	95	Reference category	Age, gender, educational level, marital status, speciality (medical vs non-medical), residential location, health insurance
						667.50	50	26	0.79 (0.42–1.49)	
						1468.63	18	14	2.54 (0.80–8.06)	
Bogale 2019 [19]	Ethiopia	General population	18 to > 60	M: 246 F: 349	Use without prescription	10.75	–	46	Reference category	Age, gender, educational level, marital status, residential location, occupational status, healthcare profession
						32.27	–	74	2.55 (1.18–5.50)	
						64.52	–	42	1.08 (0.47–2.46)	
						107.52	–	92	1.42 (0.62–3.25)	
Mate 2019 [44]	Mozambique	General population	Median (IQR): 33 (IQR: 25–47)	M:294 F:797	Use without prescription	21.24	528	108	Reference category	Age
						63.75	224	45	0.98 (0.66–1.44)	
						127.51	183	40	1.09 (0.72–1.64)	
						212.51	117	26	1.11 (0.68–1.80)	
					Incomplete course of treatment	21.24	506	150	Reference category	
						63.75	215	68	1.10 (0.78–1.55)	
						127.51	175	60	1.24 (0.86–1.79)	
						212.51	114	21	0.54 (0.32–0.89)	
Mukattash 2019 [68]	Jordan	Children´s caregivers	20 to ≥ 50	M: 134 F: 712	Use without prescription	352.50	94	41	Reference category	Age
						1058.21	325	141	0.99 (0.62–1.57)	
						1763.21	427	150	0.70 (0.44–1.10)	
Sun 2019 [69]	China	Children´s caregivers	Parents with children < 13 years old	M: 2243 F: 7283	Storage of antibiotics	230.50	2102	874	Reference category	Age, gender of the parents, gender of the child, educational level, socioeconomic characteristics (residential location and GDP per capita), health insurance, specialty (medical vs non-medical)
						615.50	2889	1434	1.22 (1.08–1.38)	
						1154.00	2749	1355	1.17 (1.02–1.33)	
						1923.00	1786	917	1.36 (1.16–1.60)	
Hu 2018 [70]	China	Medical university students	Mean (SD): 22 (1.5)	M: 661 F: 1158	Use without prescription	768.50	1565	59	Reference category	Age, gender, educational level, parents’ educational level, parents medical background, residential location, knowledge–attitudes–and practice score, center of recruitment
						2306.50	254	18	1.95 (1.13–3.36)	
Tong 2018 [71]	China	Attendants of primary care clinics	< 45 to > 60	M:340 F:374	Noncompliance	153.20	162	150	Reference category	Age, gender, educational level, residential location, occupation, employment status, knowledge about antibiotics
						344.78	180	163	0.72 (0.33–1.57)	
						651.18	187	158	0.40 (0.20–0.82)	
						880.98	185	150	0.33 (0.16–0.66)	
Peng 2018 [20]	China	University students	*Guizhou* Mean (SD): 21.3 (2.1) *Zhejiang* Mean (SD): 19.7 (2.6)	M: 2035 F: 1960	Use without prescription	230.92	–	–	Reference category	Age, socioeconomic characteristics (GDP per capita and residential location)
						1001.00	–	–	0.65 (0.39–1.09)	
						2079.08	–	–	0.66 (0.33–1.31)	
					Storage of antibiotics	230.92	–	–	Reference category	
						1001.00	–	–	1.30 (1.10–1.53)	
						2079.08	–	–	1.14 (0.90–1.43)	
					Buying without prescription	230.92	–	–	Reference category	
						1001.00	–	–	1.14 (0.90–1.44)	
						2079.08	–	–	1.05 (0.76–1.46)	
Redzick 2018 [46]	Croatia	Attendants of primary care clinics	–	M: 142 F: 402	Use without prescription	84.62	88	5	Reference category	Age
						226.12	55	13	5.14 (1.72–15.38)	
						339.32	97	4	0.71 (0.19–2.75)	
						452.52	100	15	2.93 (1.02–8.42)	
						594.02	199	25	2.39 (0.88–6.45)	
Wang 2018 [27]	China	University students	Mean (SD): 20.7 (2.7)	M: 5515 F: 5677	Storage of antibiotics	230.92	3417	–	Reference category	Age, gender, educational level, parents’ educational level, parents medical background, residential location, speciality (medical vs non-medical)
						1001.00	5823	–	1.15 (1.04–1.27)	
						2310.08	1435	–	1.02 (0.88–1.19)	
						3850.08	517	–	1.00 (0.81–1.23)	
					Use without prescription	230.92	3417	–	Reference category	
						1001.00	5823	–	0.89 (0.67–1.19)	
						2310.08	1435	–	1.13 (0.75–1.71)	
						3850.08	517	–	0.93 (0.53–1.63)	
Abdelrahman 2017 [60]	Saudi Arabia	General population	< 18 to > 65	M: 735 F: 293	Use without prescription	200.12	368	112	Reference category	Age
						867.62	146	60	1.59 (1.07–2.37)	
						2002.50	198	72	1.31 (0.91–1.88)	
						3337.63	316	146	1.96 (1.43–2.69)	
Albawani 2017 [72]	Yemen	Attendants of pharmacies	Mean (SD): 28.6 (7.7)	M: 204 F: 159	Use without prescription	116.80	268	229	Reference category	Age
						352.80	51	46	1.57 (0.59–4.19)	
						581.90	44	41	2.33 (0.69–7.89)	
Erku 2017 [73]	Ethiopia	General population	Mean (SD): 33.19 (10.82)	M: 163 F: 487	Any misuse practice	50.00	331	282	Reference category	Age, gender, educational level, marital status, employment status, household size, frequency of visiting health care institutions, satisfaction about healthcare service
						125.50	201	170	0.95 (0.58–1.55)	
						175.50	118	83	0.41 (0.25–0.68)	
Gebrekirstos 2017 [74]	Ethiopia	Attendants of pharmacies	Median (IQR): 30 (16)	M: 473 F: 307	Use without prescription	3.26	130	76	1.67 (1.13–2.48)	Age, gender, educational status, marital status, employment status, household size, residential location, type of illness, healthcare insurance, previous experience with antibiotics, access to healthcare
						13.00	92	41	0.96 (0.61–1.50)	
						26.00	81	32	0.78 (0.48–1.26)	
						39.02	477	218	Reference category	
Gillani 2017 [75]	Pakistan	Non-medical university students	Mean (SD): 23.0 (3.4)	M:352 F:375	Use without prescription	75.00	245	110	Reference category	Age, specialty (non-medical)
						225.00	180	80	0.98 (0.67–1.45)	
						400.00	136	54	0.81 (0.53–1.24)	
						600.01	166	82	1.20 (0.81–1.78)	
Hassali 2017 [76]	Malaysia	General population	Mean (SD): 28.7 (7.4)	M: 171 F: 229	Any misuse practice	124.88	231	82	Reference category	Age, gender, educational level, marital status, race, healthcare related occupation, employment status, health insurance
						499.88	94	29	0.51 (0.27–0.98)	
						1000.00	47	13	0.40 (0.16–0.78)	
						1500.13	28	7	0.42 (0.13–1.34)	
Jamhour 2017 [29]	Lebanon	General population	> 18	M: 182 F: 218	Use without prescription	499.50	88	36	Reference category	Age, gender, educational level, specialty (unrelated to health care)
						1500.00	97	54	1.81 (1.01–3.25)	
Kajeguka 2017 [77]	Tanzania	General population	Mean (SD): 35.4 (13.4)	M:144 F:156	Use without prescription	49.50	162	70	2.82 (0.47–16.68)	Age, gender, educational level, marital status, employment status, self-treated condition
						300.50	102	74	1.02 (0.22–4.76)	
						700.50	36	23	Reference category	
Kurniawan 2017 [78]	Indonesia	Attendants of primary care clinics	Median (IQR): 45 (18–49)	M: 137 F: 263	Use without prescription	87.50	186	146	Reference category	Age, gender, educational level, marital status, employment status, health insurance
						262.50	54	34	0.52 (0.24, 1.12)	
Nuñez 2017 [79]	Perú	University students	Mean: 19.82	M: 492 F: 508	Use without prescription	462.00	321	204	Reference category	Age
						1386.62	322	211	1.09 (0.79–1.51)	
						2772.62	178	119	1.16 (0.79–1.70)	
						4620.62	179	120	1.17 (0.79–1.72)	
Senadheera 2017 [80]	Sri Lanka	General population	≥ 18	M: 190 F: 174	Use without prescription	87.50	292	15	Reference category	Age, gender, educational level, employment status, health insurance, household size, receiving medical treatment in the last three months, knowledge of antibiotic name
						262.51	288	26	1.83 (0.95–3.54)	
Torres 2017 [45]	Ecuador	General population	18–64	M:97 F:110	Use without prescription	349.50	200	98	Reference category	Age
						1100.00	132	68	1.11 (0.71–1.72)	
						1775.00	36	14	0.66 (0.32–1.37)	
						2250.50	8	2	0.35 (0.07–1.76)	
Aleem 2016 [21]	Saudi Arabia	Children´s caregivers	< 25 to ≥ 55	M: 249 F: 382	Use without prescription	667.50	91	17	Reference category	Age, gender, educational level, household size
						2002.63	519	54	0.50 (0.26, 0.95)	
Bilal 2016 [81]	Pakistan	Attendants of public hospital	Mean (SD): 48.6 (4.4)	M: 263 F: 137	Use without prescription	35.00	180	172	Reference category	Age, residential location, specialty (non-medical related participants)
						105.00	73	62	0.26 (0.10–0.68)	
						210.00	49	36	0.13 (0.05–0.33)	
						415.00	36	29	0.19 (0.06–0.57)	
						685.01	62	26	0.03 (0.01–0.08)	
Zhu 2016 [82]	China	University students	18–45 (IQR: 21–22)	M: 369 F: 291	Use without prescription	40.00	45	28	Reference category	Age, gender, educational level, major, healthcare insurance, residential location
						120.08	423	192	0.50 (0.27–0.95)	
						240.08	173	83	0.56 (0.29–1.10)	
						400.08	19	13	1.32 (0.42–4.11)	
Ding 2015 [83]	China	Children´s caregivers	≤ 29 to ≥ 50	M: 70 F: 652	Noncompliance	67.08	78	15	Reference category	Age, access to healthcare (number of clinics)
						268.33	384	111	1.71 (0.93–3.13)	
						536.66	260	76	1.73 (0.93–3.24)	
Gebeyehu 2015 [84]	Ethiopia	General population	Mean (SD): *Urban* 34.1 (12.9) *Rural* 34.5 (11.5)	M:263 F:819	Any misuse practice	25.47	108	30	Reference category	Age, gender, educational level, marital status, employment status, residential location, household size Level of healthcare service satisfaction, knowledge on antibiotics use
						76.50	177	59	1.30 (0.77–2.20)	
						127.53	77	26	1.33 (0.70–2.50)	
						178.53	19	3	0.49 (0.13–1.79)	
						229.53	7	2	1.04 (0.19–5.65)	
Yousif 2015 [85]	Saudi Arabia	General population	≥ 18	M: 228 F: 172	Use without prescription	1335.00	219	173	Reference category	Age, gender, educational level, marital status, employment status, residential location
						4005.13	172	142	0.80 (0.50–1.30)	
Cheaito 2014 [86]	Lebanon	Attendants of pharmacies	Mean (SD): 38.24 (13.7)	M: 143 F: 176	Use without prescription	1000.00	278	117	Reference category	Age, gender, educational level, marital status, employment status, health insurance, having a reference doctor and frequency of consultation
						3000.00	40	17	1.02 (0.52–1.99)	
Eticha 2014 [87]	Ethiopia	University students	Mean (SD): 21 (2.06)	M: 267 F: 140	Use without prescription	6.28	159	42	Reference category	Age, gender, university year, religion, residential location
						18.92	160	38	0.87 (0.52–1.44)	
						31.56	88	32	1.59 (0.91–2.79)	
Hu 2014 [22]	Australia	General population	Mean (SD): 33 (8.2) Range: 14–63	M: 170 F: 258	Storage of antibiotics	1904.13	150	85	Reference category	Age, gender, educational level, residential location, employment status, marital status, parental status, language proficiency, main language spoken at home, health insurance
						5712.46	278	118	0.56 (0.38–0.84)	
Lv 2014 [88]	China	University students	NA	M:341 F:390	Any misuse practice	41.00	139	58	Reference category	Gender, university year, residential location, major (medical vs non-medical), health insurance
						123.08	447	175	1.14 (0.76–1.71)	
						246.08	131	56	1.00 (0.59–1.67)	
						410.08	14	5	1.26 (0.39–4.13)	
Mihretie 2014 [89]	Ethiopia	General population	Mean (SD): 37.8 (12.2)	M: 34 F: 17	Use without prescription	13.75	14	9	Reference category	Age
						38.78	10	8	2.22 (0.33–14.80)	
						67.53	10	8	2.22 (0.33–14.80)	
						102.53	14	6	0.42 (0.09–1.91)	
Shah 2014 [90]	Pakistan	University students	Mean (SD): 20.04 (1.74)	M: 253 F: 178	Use without prescription	250.00	115	51	Reference category	Age, specialty (non-medical)
						750.00	139	73	1.39 (0.85–2.28)	
						1250.00	70	38	1.49 (0.82–2.71)	
						1750.01	73	28	0.78 (0.43–1.42)	
Abobotain 2013 [61]	Saudi Arabia	Children´s caregivers	< 25 to ≥ 55	M:241 F:369	Use without prescription	667.37	91	17	Reference category	Age, educational level, marital status, household size, number of children < 12 years old, healthcare related profession
						2002.50	519	54	0.50 (0.26, 0.95)	
Pan 2012 [17]	China	University students	Mean (SD): 22.3 (2.6)	M:745 F:555	Use without prescription	38.75	548	215	Reference category	Age, gender, major, residential location, healthcare insurance
						116.33	668	352	1.73 (1.37–2.17)	
						232.58	74	46	2.54 (1.54–4.20)	
						387.58	10	8	6.20 (1.30–29.45)	
Widayati 2011 [91]	Indonesia	General population	Median (Range) *Prescribed* 40.5 (18–69) *Self-medicated* 43 (18–66)	M: 309 F: 250	Use without prescription	74.50	41	19	Reference category	Age, gender, educational level, marital status, household size, employment status, healthcare insurance
						224.50	24	15	1.93 (0.69–5.40)	
						550.00	5	1	0.29 (0.03–2.82)	
						1050.50	4	2	1.16 (0.15–9.03)	
Ilhan 2009 [16]	Turkey	Attendants of primary care clinics	Mean (SD) 39.5 (15.2)	M:1652 F:1044	Use without prescription	157.43	272	46	Reference category	Age, gender, educational level, marital status, employment status, household size, healthcare insurance (social security), perceived health status, presence of chronic diseases
						472.93	1148	188	0.96 (0.67–1.39)	
						788.43	505	107	1.32 (0.89–1.97)	
						1103.93	265	61	1.73 (1.11–2.70)	
						1419.43	350	84	1.55 (1.02–2.36)	
Hadi 2008 [92]	Indonesia	Attendants of primary care clinics	Median (range) 31 (0–87)	M: 1147 F: 1849	Use without prescription	13.50	192	30	Reference category	Age, gender, educational level, residential location, ethnicity, household size, healthcare insurance
						40.50	274	42	0.98 (0.59, 1.63)	
Al-Azzam 2007 [93]	Jordan	General population	≥ 17 to > 60	M:1040 F:1093	Use without prescription	88.75	606	204	Reference category	NA
						266.61	721	309	1.48 (1.18–1.85)	
						444.11	806	329	1.36 (1.09–1.69)	
Sawair 2007 [94]	Jordan	Attendants of primary care clinics	≤ 16 to > 65	M: 220 F: 257	Use without prescription	139.30	140	46	Reference category	Age, gender, educational level, marital status, employment status, healthcare insurance, smoking habits, self-reported health status, chronic comorbidities
						420.00	133	63	1.94 (1.18–3.21)	
						700.70	204	85	1.35 (0.85–2.14)	
Awad 2005 [95]	Sudan	General population	≤ 20 to > 60	M: 790 F: 960	Use without prescription	19.25	–	–	Reference category	Age, gender, educational level
						67.40	–	–	0.78 (0.59–1.00)	
						125.15	–	–	0.61 (0.42–0.87)	

### Income level and antibiotic misuse: continuous analysis

Overall, the data from these 57 studies were compatible with a flat linear association between income standardized to GDP per capita based on PPP and antibiotic misuse (OR 1.00; *p*-value = 0.954, *p*-value non-linearity = 0.452). Similar results were obtained for the association of income standardized to adjusted net national income per capita and antibiotic misuse (OR 1.00; *p*-value = 0.940).

**Fig. 2:**
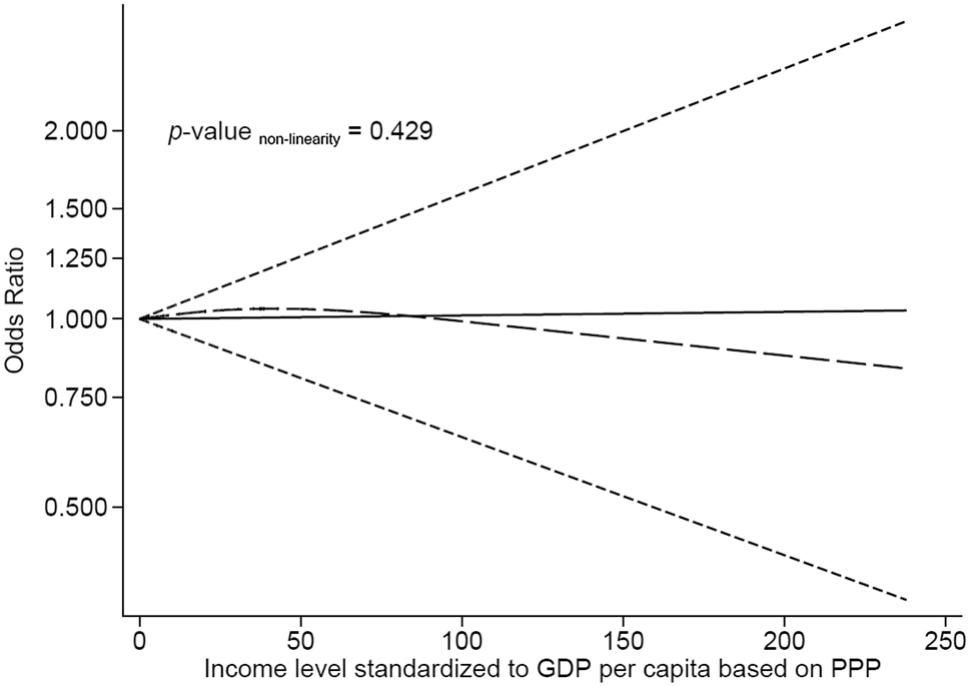
Trend of the association of income level standardized to GDP per capita based on PPP and antibiotic misuse. Solid line represents the linear trend. Long-dashed line represents the non-linear restricted cubic spline approach. Short-dashed lines represents 95% CI

As a graphical presentation of the trend, Fig. 2 shows the estimated summary odds ratio of antibiotic misuse conferred by income standardized to GDP per capita based on PPP.

### Income level and antibiotic misuse: categorical and stratified analysis

In the categorical approach of income standardized to GDP per capita based on PPP, overall, as compared to low (1st tertile), no association between income and general antibiotic misuse was observed: medium income (2nd tertile): OR 1.04; 95% CI 0.89, 1.20, and high income (3rd tertile): OR 1.03; 95% CI 0.82, 1.29 (Table 2).

**Table 2:** Meta-analysis of the association of income level represented as units of GDP per capita based on PPP with antibiotic misuse

	Number of studies	Medium income OR (95%CI)	High income OR (95% CI)
All studies	57	1.04 (0.89, 1.20)	1.03 (0.82, 1.29)
Type of misuse			
Use without prescription	43	1.06 (0.87, 1.28)	1.07 (0.84, 1.37)
Storage of antibiotics	6	1.19 (1.07, 1.32)	1.04 (0.92, 1.17)
Non-adherence	3	1.10 (0.89, 1.35)	0.49 (0.34, 0.70)
Country economy			
Low	16	1.02 (0.83, 1.24)	0.90 (0.59, 1.37)
Lower-middle	11	1.14 (0.73, 1.80)	0.92 (0.46, 1.84)
Upper-middle	25	1.17 (0.91, 1.49)	1.11 (1.00, 1.22)
High	5	0.90 (0.44, 1.85)	1.04 (0.33, 3.28)
WHO Region			
African	14	1.18 (1.00, 1.39)	0.96 (0.67, 1.38)
Eastern Mediterranean	17	0.92 (0.65, 1.32)	0.95 (0.58, 1.57)
South-East Asian	6	1.11 (0.62, 2.00)	1.53 (0.81, 2.92)
Western Pacific	16	0.99 (0.82, 1.20)	1.05 (0.92, 1.19)
Survey year			
Until 2015	29	0.95 (0.75, 1.20)	0.91 (0.62, 1.35)
After 2015	28	1.12 (0.99, 1.26)	1.15 (0.93, 1.41)
Literacy rate			
< 90%	20	1.03 (0.82, 1.29)	1.02 (0.68, 1.54)
≥ 90%	37	1.09 (0.93, 1.28)	1.02 (0.84, 1.23)
Pre-tested or validated questionnaire			
No	10	1.02 (0.51, 2.06)	0.90 (0.34, 2.36)
Yes	47	1.06 (0.91, 1.24)	1.04 (0.85, 1.27)
Adjustment			
Incomplete	47	1.09 (0.95, 1.24)	1.05 (0.84, 1.31)
Complete	10	0.90 (0.71, 1.15)	0.60 (0.30, 1.23)
Quality Score			
Lower quality (≤ 3 points)	24	0.99 (0.75, 1.31)	1.09 (0.72, 1.66)
Higher quality (> 3 points)	33	1.04 (0.86, 1.25)	1.03 (0.81, 1.31)

Stratified analysis revealed that medium income was associated with 19% higher odds of *storage of antibiotics* (OR 1.19; 95% CI 1.07, 1.32),); nonetheless, we did not observe any significant association between high income and this type of misuse (OR 1.04; 95% CI 0.92, 1.17). It is noteworthy to mention that *storage of antibiotics* was evaluated in five studies carried out in China [20, 27, 28, 43, 44] and in a sixth study that was undertaken in Australia but involved Chinese immigrants [22]. High income was associated with 51% lower odds of *non-adherence to antibiotics* treatment (OR 0.49, 95% CI 0.34, 0.70) (Table 2). When restricting the analysis to low-wealth countries, high- income individuals were at 11% higher odds of antibiotic misuse than those with low income in upper–middle wealth countries (OR 1.11; 95% CI 1.00, 1.22) (Table 2). Our findings also suggested an association between medium-income medium income level and antibiotic misuse in African countries (OR 1.18; 95% CI 1.00, 1.39) (Table 2). After 2015, the odds of misuse of antibiotics in medium- income individuals increased when compared with studies undertaken until 2015 (OR_until 2015_ 0.95; 95% CI 0.75, 1.20 and OR_after 2015_ 1.12; 95% CI 0.99, 1.26). Similar findings were obtained for high- income individuals (OR_until 2015_ 0.91; 95% CI 0.62, 1.35 and OR_after 2015_ 1.15; 95% CI 0.93, 1.41) (Table 2). No meaningful difference in the odds of antibiotic misuse by medium- and high- income individuals was observed when countries were grouped according to literacy rate (Table 2).

The categorical approach of income standardized to net national income per capita showed similar results to that of income standardized to GDP per capita based on PPP (data not shown).

### Methodological characteristics of the studies

Restricting the analysis to those studies that used pretested or validated questionnaires did not yield any substantial modification in the pooled OR estimates (OR_medium_ 1.06; 95% CI 0.91, 1.24 and OR_high_ 1.04; 95% CI 0.85, 1.27) (Table 2).

Studies that incompletely controlled for sex, age, educational level and household size showed higher pooled estimates than those with complete control of those variables in medium income (OR_incomplete_ 1.09; 95% CI 0.95, 1.24 and OR_complete_ 0.90; 95% CI 0.71, 1.15) and in high income (OR_incompletz_ 1.05; 95% CI 0.84, 1.31 and OR_complete_ 0.60; 95% CI 0.30, 1.23) (Table 2).

No notable difference was observed between pooled estimates from studies with lower-quality (≤ 3 points) and those from studies with higher-quality score (> 3 points) (Table 2).

### Publication bias

**Fig. 3:**
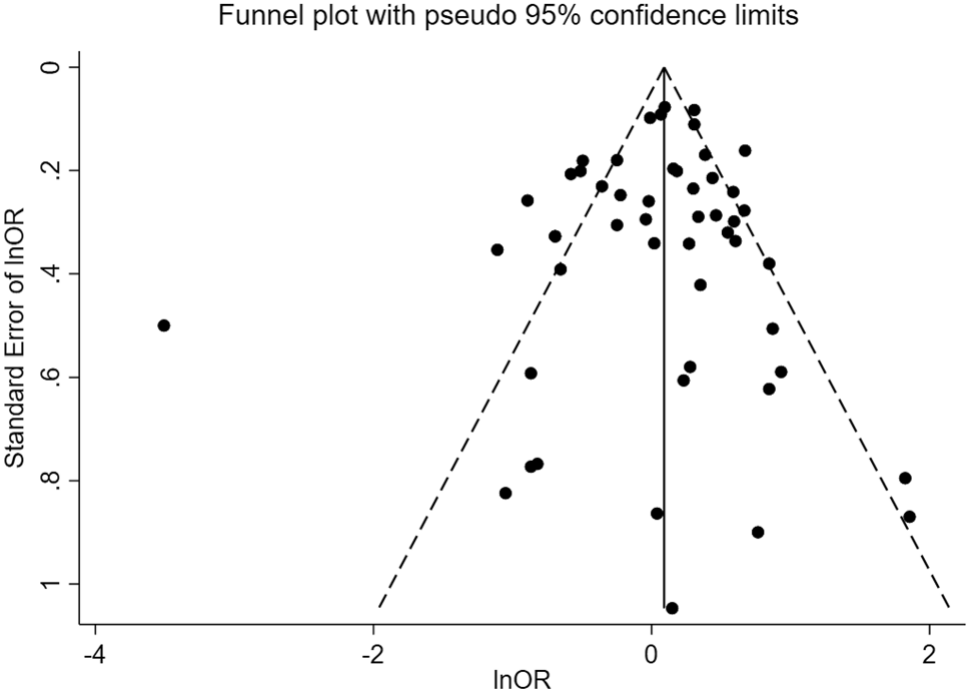
Funnel plot of studies about income and antibiotic misuse

The funnel plot (Fig. 3) and Egger’s test of the null hypothesis (*p*-value = 0.39) did not suggest evidence of publication bias. These findings were further confirmed by the Trim-and-Fill analysis that did not yield to the addition of any study.

## Discussion

Antibiotic resistance is an internationally growing multifaceted emergency that has been exacerbated by antibiotic misuse and has left devastating impact at the clinical, health and socio-economic levels. If not controlled, antibiotic resistance will convert into the major cause of death in 2050 [8].

To the best of our knowledge, this is the first meta-analysis that assesses the dose–response association between income level and misuse of antibiotics. Our results agree well with the hypothesis of no association between income level and misuse of antibiotics. Subgroup analyses reveal a dose–response association of medium- and high- income levels with specific types of antibiotic misuse, i.e., storage of drug leftover and non-adherence, country wealth, geographic region and study period.

Our primary findings suggest that the odds of misuse of antibiotics do not differ between poor and wealthy people. This is in line with the fact that both low- and high- income individuals tend to self-medicate. On the one hand, under constrained financial resources, especially in less developed economies where access to health facilities is limited, self-medication is the only available option of healthcare [47]. By self-medicating, individuals with low income avoid expenses of medical consultation and subsequent lab tests. Low- income households report forgone care more often than those with high- income level [48]. They often cut -back basic needs and take less medication than prescribed, due to cost [49, 50], explaining therefore the observed higher likelihood of adherence to treatment by high- income than by low- income individuals. On the other hand, people with high- income level tend to medicate themselves as they have easier access to sources of information including internet to seek health information [51], can afford purchasing non-reimbursed medicines, and have more social support that increases their access to unprescribed medicines including through sharing with families and friends [52].

Our dose–response meta-analysis also showed that medium- income individuals have higher odds of storing antibiotic leftover than those with low income. This could be related to higher financial affordability by medium-income medium income individuals to purchase and store antibiotics. Our results also show a higher likelihood of misuse by high-income individuals in upper–middle wealth countries. Consistent with our findings, an earlier report about the economy of self-medication in general, indicated that the demand for self-medication declines with rising the income level of high- income individuals, but increases with increasing the income of low-income individuals, resulting in a null pooled effect between income and self-medication [47].

We also reported that medium- income individuals in Africa have higher chances of antibiotic misuse, probably due to the poor enforcement of antibiotic dispensing regulations in those regions.

We observed a marginal increase in the odds of misuse of antibiotics by medium- income and high- income individuals after 2015 than before this period. This could be related to two main motives;: first, as concluded by WHO in its report *Global Spending on Health*, the expenditure on health is growing faster than economies, leading to a doubling of the out-of-pocket spending and very large differences between high- and low-wealth countries concerning health expenditure [53], second, not all countries have developed and implemented sufficient measures to control the dispensing of antibiotics, and thus people with greater financial resources continued using antibiotics without prescription. A recent review report indicated that more than half of the antibiotics worldwide are dispensed without prescription [54]. Consequently, the WHO placed a new urgent call to control antibiotics resistance crisis on 2019 [55].

The findings of this meta-analysis are unlikely to be affected by publication bias as revealed by the negative result of Egger’s test and the trim-and-fill analysis that did not suggest imputation of any additional study.

This meta-analysis suffers from several limitations. All eligible studies were of cross-sectional design, which, theoretically, limited any causal inference. However, income is a relatively stable variable through time and, which mitigates this limitation. Furthermore, only one-fifth of included studies performed a complete control for socio-demographic variables, and higher OR estimates were obtained from studies with incomplete adjustment than in studies with complete adjustment. This reveals that our findings could be overestimated due to incomplete adjustment. Additional studies that control adequately for all potentially related socio-demographic variables are needed to confirm our results. Also, one-sixth of studies did not employ a pretested or validated questionnaire to ascertain the exposure and the outcome. However, this was unlikely to affect our results as constraining the analysis to the remaining studies did not introduce any change in the overall effect.

Our analysis was based on random-effect models to account for heterogeneity between studies [56–58]. Heterogeneity was expected in our study due to difference in the defined levels of income, period of antibiotic use (for example, use in the past month [59], past 3 months [60] and past year [61]), and settings. Experts in meta-analysis emphasize that heterogeneity is the expectation in any meta-analysis rather than the exception [62] and that no amount of heterogeneity is considered unacceptable as long as the inclusion criteria are clearly defined and the data are correctly analysed [56].

Understanding the socioeconomic properties of antibiotic misuse is crucial to develop related intervention programs and health policies, yet addition of high-quality studies that control for socio-demographic and socio-economic indicators are needed to confirm our findings.

## Data Availability

The data generated and analyzed in the meta-analysis are included in the article. The data are available by accessing the cited references.
